# Retrospective study on the treatment of osteoporotic vertebral compression fractures with alfacalcidol and percutaneous kyphoplasty

**DOI:** 10.3389/fsurg.2026.1745068

**Published:** 2026-07-06

**Authors:** Sihua Jin, Zhidong Wang, Haixiang Quan, Hongming Zhou, Donghua Chen, Chunhong Jin, Chao Chen

**Affiliations:** 1 Department of Orthopaedic Surgery, The Fifth People's Hospital of Wujiang Area, Suzhou, Jiangsu, China; 2 Department of Orthopaedic Surgery, The First Affiliated Hospital of Soochow University, Suzhou, Jiangsu, China

**Keywords:** alfacalcidol, bone metabolism, osteoporosis, osteoporotic vertebral compression fractures, percutaneous kyphoplasty

## Abstract

**Objective:**

To evaluate the clinical efficacy of *percutaneous kyphoplasty* (PKP) combined with alfacalcidol in the treatment of osteoporotic vertebral compression fractures (OVCF), and to explore the impact on bone metabolism and functional recovery.

**Methods:**

This retrospective study included 120 patients with single-level OVCFs who underwent PKP between January 2021 and January 2023. Patients were divided into two groups: the basic treatment group (BT, *n* = 60) received standard postoperative care including calcium and vitamin D supplementation; the alfacalcidol group (AFC, *n* = 60) received additional oral alfacalcidol. Clinical parameters—including bone mineral density (BMD), bone metabolism markers (NMID, P1NP, β-CTX), Visual Analog Scale (VAS) for pain, and Japanese Orthopaedic Association (JOA) scores—were recorded preoperatively, and at 6 and 12 months postoperatively.

**Results:**

Both groups showed significant postoperative improvement in BMD, VAS, and JOA scores. However, the AFC group demonstrated greater increases in BMD and more pronounced reductions in bone turnover markers at both follow-up points (*P* < 0.05). Additionally, pain relief and functional recovery were significantly better in the AFC group. No serious adverse events were observed.

**Conclusion:**

PKP combined with alfacalcidol is more effective than PKP alone in improving bone metabolism, relieving pain, and promoting functional recovery in patients with OVCF. This combination therapy addresses both the structural and metabolic aspects of osteoporosis and may help reduce future fracture risk.

## 1 Introduction

Osteoporosis is a systemic skeletal disorder characterized by reduced bone mass and microarchitectural deterioration of bone tissue, leading to increased bone fragility and susceptibility to fractures (1). Among osteoporotic fractures, vertebral compression fractures (OVCF) are the most common, especially in the elderly population (2). These fractures often occur as a result of minimal trauma or even spontaneously, causing acute back pain, decreased mobility, spinal deformity, and a decline in quality of life (3). Moreover, OVCF are associated with a higher risk of subsequent fractures and increased mortality. Despite their clinical significance, OVCF are frequently underdiagnosed or inadequately treated, highlighting the need for effective therapeutic strategies that not only stabilize the fracture but also address the underlying bone fragility (4).

The management of OVCF typically involves a combination of conservative, surgical, and pharmacological approaches, depending on the severity of symptoms and the patient's overall condition (5). Conservative treatment includes bed rest, analgesics, bracing, and physical therapy, which may offer symptomatic relief but often fail to restore vertebral height or prevent further collapse (6). Percutaneous kyphoplasty (PKP), a minimally invasive procedure, has emerged as an effective surgical option that stabilizes the fracture, restores vertebral body height, and provides rapid pain relief (7).

Vitamin D plays a fundamental role in calcium and phosphate homeostasis and is essential for maintaining bone health (8). In osteoporotic patients, especially the elderly, vitamin D deficiency is common and contributes to impaired calcium absorption, secondary hyperparathyroidism, and increased bone turnover, thereby accelerating bone loss and fracture risk (9). Vitamin D analogs, such as alfacalcidol, are frequently used to correct these deficiencies and improve bone metabolism (10). Alfacalcidol is a prodrug that is converted in the liver to the active form, calcitriol, bypassing the need for renal hydroxylation (11). This makes it particularly suitable for elderly patients or those with compromised renal function.

Clinical studies have shown that alfacalcidol not only enhances calcium absorption and bone mineralization but also improves muscle strength and balance, thereby reducing the risk of falls and subsequent fractures (10). When used in combination with surgical treatments like PKP, alfacalcidol may support vertebral healing and help prevent future osteoporotic fractures by addressing the underlying metabolic imbalance. Therefore, we retrospectively studied the patients with OVCF to evaluate the clinical efficacy and safety of alfacalcidol combined with PKP.

## 2 Methods

### 2.1 Patient selection

Patients treated with PKP in our hospital were selected in this retrospective study from January 2021 to December 2023. All radiologic data and clinical outcomes were collected from the medical record. The inclusion criteria were as follows: (1) diagnosed as primary or glucocorticoid-induced osteoporosis; (2) low bone mineral density (BMD) with T-value ≤−2.5; (3) experienced vertebral compression fracture received PKP; (4) received a persistent, subsequent anti-osteoporosis treatment, including alfacalcidol or basic treatment of calcium and vitamin D supplementation after PKP.

Patients were excluded when: (1) underwent PKP due to pathological fracture; (2) combined with other internal diseases, such as osteogenesis imperfecta, hypercalcemia or parathyroid diseases; (3) received alfacalcidol before PKP or other anti-osteoporosis treatment after surgery, including bisphosphonates, estrogen, calcitonin and selective estrogen receptor modulators.

Overall, 120 patients with OVCF, who received PKP in our hospital, were divided into alfacalcidol (AFC) group (*n* = 60) and basic treatment (BT) (*n* = 60) group based on the anti-osteoporosis strategy after PKP.

Informed consent about reviewing the medical records was obtained from each patient. This retrospective study was in accordance with the principles of the Declaration of Helsinki.

### 2.2 Surgical procedures

All patients performed BMD, x-ray, CT scan and magnetic resonance imaging (MRI) examination of the spine before surgery. Patients were positioned prone on the operating table. After local anesthesia, a probe was placed into the vertebral through a small incision. The bone was drilled, and a balloon was inserted on each side and inflated with the contrast medium to achieve the satisfactory height recovery. Under the monitor of fluoroscopic, the bone cement was injected to the vertebra carefully.

### 2.3 Treatment protocol

The present study only evaluated alfacalcidol as the targeted pharmacological intervention, while first-line anti-osteoporosis medications including bisphosphonates, denosumab, and teriparatide were excluded. This exclusion was intentional to eliminate confounding from multiple antiresorptive or anabolic agents and to clearly determine the independent effect of alfacalcidol when combined with PKP. Although real-world osteoporosis management often uses combination regimens, our focused protocol supports the role of alfacalcidol as a valuable adjunctive therapy, especially in patients unsuitable for or intolerant to standard first-line agents.

The cost and hesitation about its effectiveness were the main reasons that patients didn't receive alfacalcidol. All patients received daily supplements of 1,000 mg calcium and 400–1,200 vitamin D as the strategy of basic treatment. Additionally, patients in AFC group received alfacalcidol 1μg/d (approximate 10 drops).

### 2.4 Evaluation indicators

The BMD value, serum bone metabolic indices, visual analog scale (VAS) score, Japanses orthopeadic association (JOA) score and adverse reactions before and 6 and 12 months after surgery were evaluated. The BMD values of the left femoral neck were evaluated with the dual-energy x-ray. The serum indices of N-terminal molecular fragment (NMID), the peptide type I collagen amino end (P1NP) and beta collagen degradation product (β-CTX) levels were determined by providing manufacturing bone metabolism. VAS score and JOA score were recorded to evaluated the pain and quality of life. Additionally, adverse events including nausea, vomiting, fatigue and new fracture of the patients were observed.

### 2.5 Statistical analysis

The data was analyzed using SPSS 19.0. The results are presented as means ± SD. Student's t test and one way ANOVA analysis were applied for continuous data, and chi-square test for categorical data. A *p* value of < 0.05 was considered for statistical significance.

## 3 Results

### 3.1 Demographic characteristics

The baseline characteristics of all patients were summarized in Table 1. There consists of 46 males and 74 females, with the mean age of 70.90 ± 6.48. According to the type of anti-osteoporosis strategy, 60 patients were assigned to the AFC group and 60 to the BT group. The characteristics of the patients in two groups had no significant difference.

**Table 1:** The baseline characteristics of patients in two groups.

Item	AFC group *N* = 60	BT group *N* = 60	*P*
Age	71.22 ± 6.87	70.32 ± 7.01	0.479
Male/female	21/39	25/35	0.453
Location			0.577
Thoracic vertebra	26	23	
Lumbar vertebra	34	37	
BMD	−3.09 ± 0.34	−3.02 ± 0.34	0.244
VAS score	6.53 ± 1.08	6.72 ± 1.11	0.360
JOA score	15.12 ± 2.46	15.20 ± 2.70	0.860
NMID/μg/L	21.22 ± 5.09	20.52 ± 5.35	0.465
P1NP/μg/L	40.09 ± 2.05	39.86 ± 1.90	0.534
β-CTX/μg/L	0.56 ± 0.13	0.55 ± 0.14	0.696

### 3.2 Clinical outcomes

#### 3.2.1 Bone mineral density

**Figure 1:**
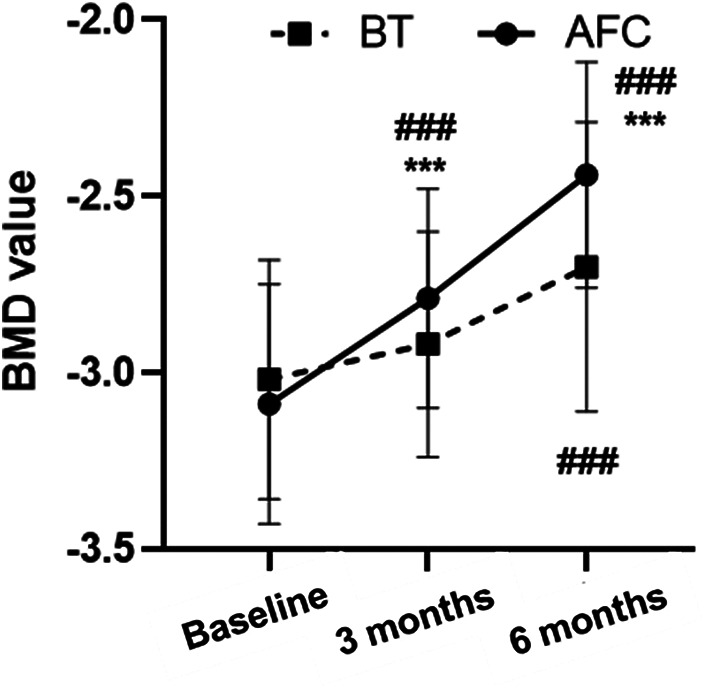
The BMD values in the two groups. (#, compared to the baseline, #*p* < 0.05, ##*p* < 0.01, ###*p* < 0.001; *, compared to the BT group, *p < 0.05, **p < 0.01, ***p < 0.001).

Patients were diagnosed as osteoporosis based on the significant decrease of BMD of the left femoral neck. In this study, the baseline of BMD was similar between the two groups (*p* = 0.244). In the BT group, BMD at 6 and 12 months was −2.80 ± 0.42 and −2.70 ± 0.41, respectively. Compared to the baseline, the difference was only observed in BMD at 12 months (*p* < 0.001). In the AFC group, the BMD at 6 and 12 months after treatment was −2.79 ± 0.31 and −2.44 ± 0.32 respectively, revealing a significant increase compared to the baseline (*p* < 0.001, *p* < 0.001). Additionally, the BMD in AFC group was significantly higher than that in the BT group at 6 and 12 months (*p* = 0.028; *p* < 0.001) after treatment (Figure 1).

#### 3.2.2 Serum bone metabolic indices

**Figure 2:**
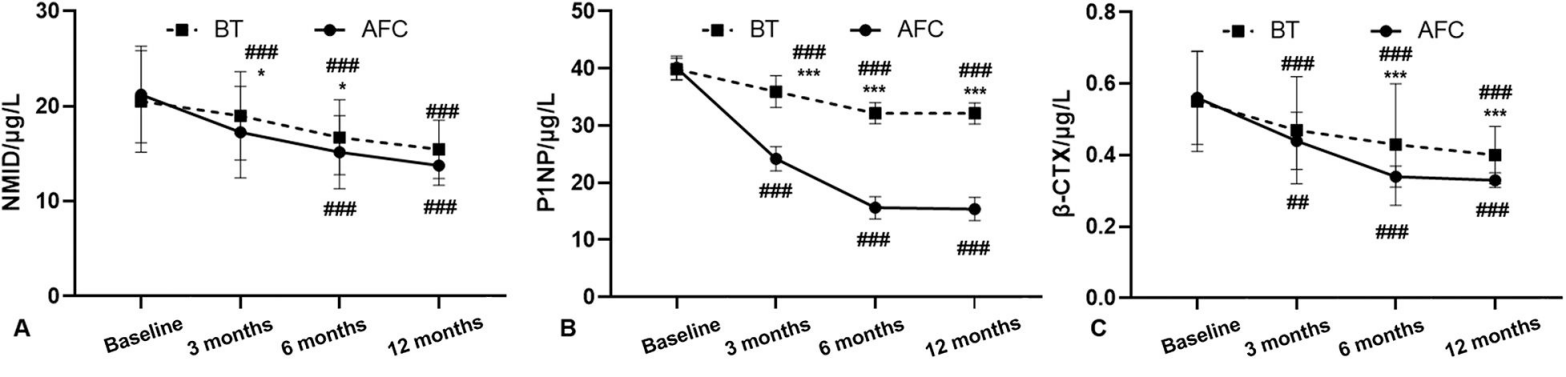
**(A)** The NMID values in the two groups; **(B)** The P1NP values in the two groups; **(C)** The β-CTX values in the two groups. (#, compared to the baseline, #*p* < 0.05, ##*p* < 0.01, ###*p* < 0.001; *, compared to the BT group, *p < 0.05, **p < 0.01, ***p < 0.001).

Serum makers NMID, P1NP, and β-CTX were used to evaluate bone absorption. The baseline of NMID, P1NP, and β-CTX in AFC group was 20.52 ± 5.35 μg/L, 39.86 ± 1.90 μg/L, and 0.55 ± 0.14 μg/L, which had no significant difference compared the indices in BT group. After anti-osteoporosis treatment, the three serum makers in two groups were significantly lower than the baseline, and the indices in AFC group was lower than that in BT group (Figure 2A–C).

#### 3.2.3 VAS score

**Figure 3:**
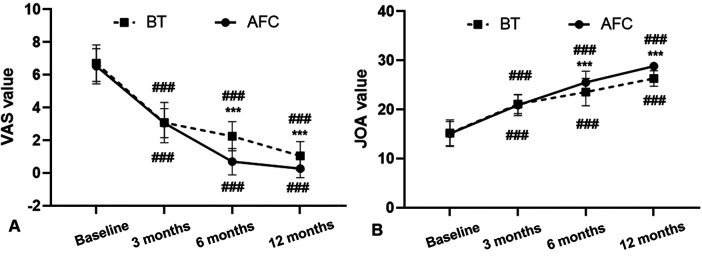
**(A)** The VAS values in the two groups; **(B)** The JOA values in the two groups. (#, compared to the baseline, #*p* < 0.05, ##*p* < 0.01, ###*p* < 0.001; *, compared to the BT group, *p < 0.05, **p < 0.01, ***p < 0.001).

Before surgery, the mean VAS scores in the BT group and AFC group were 6.53 ± 1.08 and 6.72 ± 1.11, respectively, with no significant difference (*p* = 0.865). Immediately after surgery, both groups showed a significant reduction in VAS scores compared to baseline. The mean VAS score in the BT group decreased to 3.08 ± 1.23 (*p* < 0.001), and in the AFC group, it decreased to 3.05 ± 0.89 (*p* < 0.001). At 6-month after treatment, the VAS scores in the AFC group continued to decline to 0.70 ± 0.81, which was significantly lower than that in the BT group (*p* < 0.001). This difference in pain scores between the two groups was maintained at 12 months (*p* < 0.001) after treatment, indicating better long-term pain relief in the AFC group (Figure 3A).

#### 3.2.4 JOA score

The JOA score was used to assess functional recovery. As shown in Table 1, the baseline JOA scores in BT group and AFC group were 15.20 ± 2.70 and 15.12 ± 2.46, respectively, with no significant difference. Post-operatively, both groups showed a significant improvement in JOA scores. At 6 months post-operation, the mean JOA score in the AFC group was 25.55 ± 2.30, which was significantly lower than that in BT group (23.58 ± 2.80, *p* < 0.001), suggesting better functional recovery in the combination treatment group. This difference was also observed at 12 months post-operation (*p* < 0.001) (Figure 3B).

#### 3.2.5 Adverse events

Cement leakage was observed in 8 cases in AFC group and 10 in BT group. In the AFC group, adverse events occurred during the administration of alfacalcidol in 3 cases, including gastrointestinal reaction and pruritus.

## 4 Discussion

This retrospective study demonstrated that combining alfacalcidol with PKP provides superior clinical outcomes in patients with OVCF. Both groups experienced significant pain relief and functional improvement postoperatively; however, the AFC group showed greater and more sustained benefits. BMD increased more significantly in the AFC group at both 6 and 12 months, suggesting enhanced bone remodeling. Serum markers of bone turnover (NMID, P1NP, β-CTX) decreased in both groups, with greater reductions in the AFC group, indicating better metabolic balance. JOA scores confirmed superior pain control and functional recovery in the AFC group, likely due to improved bone quality and reduced risk of microfractures. The incidence of complications, including cement leakage, was similar between groups, confirming the safety of the combined approach. These findings highlight that alfacalcidol not only supports bone metabolism but also enhances long-term outcomes following PKP. Therefore, integrating vitamin D analog therapy into the perioperative management of OVCF may improve patient recovery and reduce future fracture risk. However, the superior VAS reduction in the AFC group should be interpreted cautiously. Alfacalcidol has no direct analgesic effect, and early pain relief after PKP mainly results from vertebral stabilization and mechanical support. The long-term difference in pain scores is more likely attributable to indirect effects, including improved bone mineral density, reduced vertebral micro-instability, better functional recovery, and potentially unmeasured differences in rehabilitation or patient characteristics, rather than a direct pharmacological analgesic action.

Alfacalcidol exerts its anti-osteoporotic effects by enhancing intestinal calcium absorption, regulating bone turnover, and improving bone mineral density, which supports its adjunctive role after PKP. While alfacalcidol offers clear benefits in bone metabolism through enhanced intestinal absorption of calcium and phosphate, its clinical use requires careful attention to potential adverse effects (12). The most clinically significant concern is hypercalcemia, which may arise as a direct consequence of its pharmacological action. Mild hypercalcemia often presents with nonspecific symptoms such as nausea, vomiting, constipation, and loss of appetite—manifestations that can be easily overlooked in elderly patients with multiple comorbidities (13). More severe cases may progress to confusion, intense thirst, polyuria, muscle weakness, and, in extreme situations, life-threatening cardiac arrhythmias or acute kidney injury (14). Prolonged hypercalcemia may also contribute to vascular and soft tissue calcification, which is particularly concerning in elderly patients with pre-existing cardiovascular or renal conditions (15). In the present study, no cases of symptomatic hypercalcemia or severe adverse events were observed, and reported reactions were limited to mild gastrointestinal discomfort and pruritus in three patients, none of which required discontinuation of therapy. This favorable safety profile aligns with previous reports indicating that alfacalcidol at 1 μg/day is generally well tolerated. However, this finding should be interpreted cautiously, as routine biochemical monitoring was not systematically performed at every follow-up, and subclinical hypercalcemia may have gone undetected.

The findings of this study are consistent with previous literature supporting the use of PKP as an effective intervention for pain relief, vertebral height restoration, and functional improvement in patients with OVCF (16–18). Numerous studies have reported significant reductions in VAS scores and improvements in the Oswestry Disability Index (ODI) following PKP, similar to the outcomes observed in our cohort (19). In terms of pharmacological management, the beneficial role of alfacalcidol in osteoporosis has also been well documented (20). For instance, several randomized controlled trials have demonstrated that Alfacalcidol improves BMD and reduces the incidence of new vertebral and non-vertebral fractures, particularly in elderly populations (21). Compared with native vitamin D supplements like cholecalciferol, Alfacalcidol has shown superior efficacy in patients with impaired renal function or low baseline vitamin D activation (11, 22). Our results align with these findings, indicating that the addition of Alfacalcidol to PKP may offer synergistic benefits by addressing both the mechanical and metabolic components of OVCFs. However, few studies have specifically investigated this combined approach, highlighting the novelty and clinical relevance of our retrospective analysis.

This study has several important limitations. First, the non-randomized retrospective design introduced potential selection bias, as treatment allocation was based on patient preference and socioeconomic factors rather than clinical indications, and no multivariate or propensity score adjustment was performed. Second, the between-group difference in VAS scores should be interpreted cautiously because alfacalcidol has no direct analgesic effect; long-term pain improvement is likely related to better bone metabolism and functional recovery rather than a direct pharmacological pain-relieving action. Third, only alfacalcidol was investigated while standard first-line anti-osteoporosis agents were excluded, which may limit the generalizability to real-world clinical practice. Fourth, key clinical outcomes including new vertebral fracture and refracture rates were not collected, the sample size was relatively modest, the 12-month follow-up may be insufficient to assess long-term efficacy, and patients may have heterogeneous responses to treatment.

## 5 Conclusion

In summary, the combination of PKP and alfacalcidol significantly improves clinical and radiological outcomes in patients with OVCFs. Adjunctive alfacalcidol therapy led to greater improvements in bone mineral density, reduced bone turnover markers, enhanced pain relief, and better functional recovery. These findings suggest that integrating vitamin D analogs into the perioperative management of OVCF addresses both the mechanical and metabolic aspects of the disease, potentially reducing the risk of future fractures.

## Data Availability

The original contributions presented in the study are included in the article/Supplementary Material, further inquiries can be directed to the corresponding author.
